# Increased risk for urological cancer associated with anxiety disorder: a retrospective cohort study

**DOI:** 10.1186/s12894-016-0187-x

**Published:** 2016-11-16

**Authors:** Yung-Chan Chen, Li-Ting Kao, Herng-Ching Lin, Hsin-Chien Lee, Chung-Chien Huang, Shiu-Dong Chung

**Affiliations:** 1 Department of Psychiatry, Shuang Ho Hospital, Taipei Medical University, New Taipei City, Taiwan; 2 Graduate Institute of Life Science, National Defense Medical Center, Taipei, Taiwan; 3 Sleep Research Center, Taipei Medical University, Taipei, Taiwan; 4 Department of Psychiatry and Medical Humanities, School of Medicine, College of Medicine, Taipei Medical University, Taipei, Taiwan; 5 School of Health Care Administration, Taipei Medical University, Taipei, Taiwan; 6 Division of Urology, Department of Surgery, Far Eastern Memorial Hospital, New Taipei City, Taiwan; 7 Graduate Program in Biomedical Informatics, College of Informatics, Yuan-Ze University, Chung-Li, Taiwan; 8 Department of Surgery, Far Eastern Memorial Hospital, No.21, Sec. 2, Nanya S. Rd., Banciao Dist, New Taipei City, 220 Taiwan

**Keywords:** Anxiety disorder, Urological cancer, Bladder cancer, Epidemiology

## Abstract

**Background:**

Anxiety disorders (ADs) are common with a high rate of medical comorbidities. Although the association between ADs and the overall cancer risk remains controversial, patients with ADs were found to be more likely to develop specific cancer types. Herein, we estimated the risk of developing urological cancers among patients with ADs in a 5-year follow-up period using a population-based database.

**Methods:**

Two study cohorts were identified from the Taiwan Longitudinal Health Insurance Database 2005: patients with ADs, and comparison subjects selected by one-to-one matching for sex, age, and the year of recruitment. Follow-up was undertaken to determine whether sampled patients and comparison subjects had developed urological cancers in the subsequent 5 years.

**Results:**

We found that urological cancers occurred among 0.54% of patients with ADs and 0.13% of comparison subjects. After adjusting for sociodemographic characteristics, medical comorbidities, and alcohol and tobacco use disorder, the stratified Cox proportional hazard regression suggested that patients with ADs were more likely to develop urological cancers relative to comparison subjects (adjusted hazard ratio, 3.67; 95% confidence interval, 2.85 ~ 4.72). The adjusted HR for males with ADs was 3.82 (95% CI: 2.79 ~ 5.23) in comparison to males without ADs. In addition, the adjusted HR for females with ADs was 3.47 (95% CI: 2.26 ~ 5.31) than those females without ADs.

**Conclusions:**

We concluded that during the 5-year follow-up period, there was a significantly increased risk of urological cancers among patients with ADs.

## Background

Anxiety disorders (ADs), including panic disorder, generalized anxiety disorder, phobic disorder and so on, are common mental disorders within a disease category characterized by excessive worry which might affect 9.2% ~ 28.7% of the general population around the world [1, 2]. The prevalence of ADs is not only high but also strikingly increasing in Asian populations [3, 4].

ADs are shown to markedly compromise the quality of life and psychosocial functioning in several domains [5]. ADs are highly comorbid with numerous chronic medical illness including diabetes, coronary artery disease, congestive heart failure, asthma, chronic obstructive pulmonary disease, and arthritis [6, 7]. Thus, the research focus has gradually turned to the relationship between ADs and malignancies. Indeed, psychological distress was proven to have an adverse effect on cancer survival [8, 9].

Nevertheless, it is still controversial and unclear whether the existence of ADs is associated with the subsequent occurrence of cancer [8, 10–13]. Patients with ADs were found to be at increased risks of developing specific cancer types including lung, brain, and prostate cancers [11–13]. Although the underlying mechanism remains obscure, indirect relationships through a surveillance bias and unhealthy lifestyle behaviors were proposed to interpret this association [12, 13]. It is debatable whether these factors could contribute to the overall cancer risk instead of to certain cancer types such as prostate and other urological cancers found in previous studies.

It is possible that the weak, though significant, association seen in previous studies lends support to a direct relationship between ADs and urological cancers. Even so, there is evidence that dysfunction of the serotonin receptor pathway, crucial in anxiety disorders, might also be involved in the proliferation of urological cancer [14, 15]. This could pose a threat to both a patient's mental and physical health. Considering the biological plausibility, this study aimed to investigate the relationship between ADs and the subsequent occurrence of urological cancers using a longitudinal population-based database.

## Methods

### Database

The data for this retrospective cohort study were sourced from the Taiwan Longitudinal Health Insurance Database 2005 (LHID2005). The LHID2005, compiled by the Taiwan National Health Research Institute, consists of original medical claims data for 1,000,000 individuals randomly sampled from all enrollees (*n* = 25.68 million) in the Taiwan National Health Insurance (NHI) program in 2005. Since around 96% of the Taiwanese population enrolled in the NHI program, the LHID2005 provides an exclusive opportunity for researchers to follow-up the use of all medical services for nationwide, population-based sampled subjects. Hundreds of studies utilizing this dataset have been published in internationally peer-reviewed journals [16].

This study was exempt from full review by the Institutional Review Board of National Defense Medical Center since the LHID2005 consists of de-identified secondary data released to the public for research purposes.

### Study sample

**Fig. 1:**
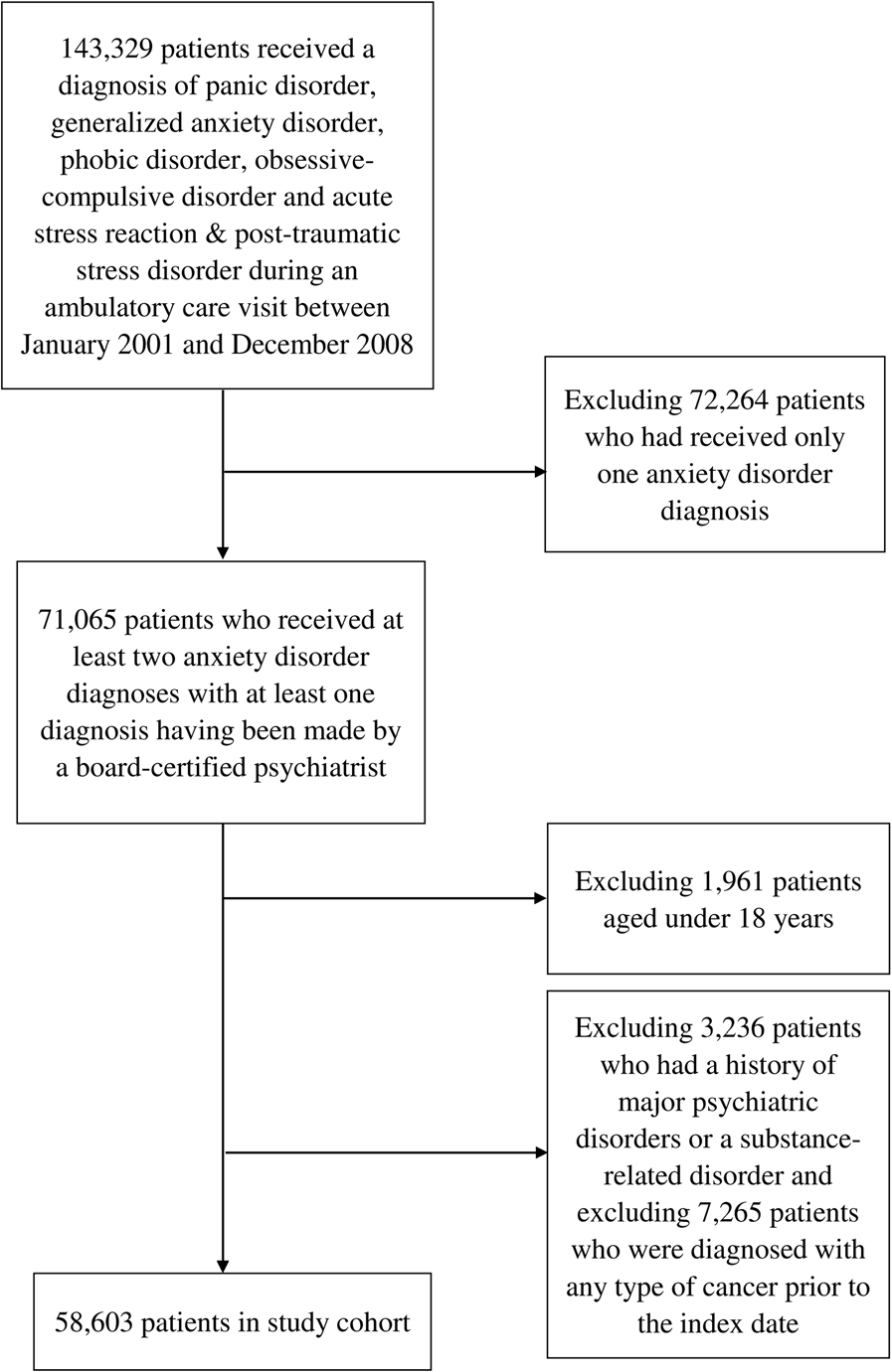
Flow diagram for study cohort

This study features a study cohort and a comparison cohort. The study cohort included all patients who had received one of the following ADs diagnoses between January 1, 2001 and December 31, 2008: panic disorder (ICD-9-CM: 300.01), generalized anxiety disorder (ICD-9-CM: 300.02), phobic disorder (ICD-9-CM: 300.2, 300.20 ~ 300.29), obsessive-compulsive disorder (ICD-9-CM: 300.3) and acute stress reaction & post-traumatic stress disorder (ICD-9-CM: 308, 309.81 ~ 309.83) (*n* = 143,329). In order to increase the diagnosis validity, this study only included patients with ADs who received at least two anxiety disorder diagnoses with at least one diagnosis having been made by a board-certified psychiatrist (*n* = 71,065). We assigned the date of the second medical service utilization for ADs during the recruitment period as the index date. We then excluded patients aged under 18 years (*n* = 1,961) in order to limit the study to the adult population. Additionally, subjects who had a history of major psychiatric disorders or a substance-related disorder (ICD-9-CM codes 290 ~ 299 or 303 ~ 305) (*n* = 3,236) were excluded in order to exclude possible confounding effects of other mental illnesses on the association between ADs and urological cancers. We further excluded those subjects who were diagnosed with any type of cancer (ICD-9-CM codes 140 ~ 239) prior to the index date (*n* = 7,265). As a result, 58,603 subjects with ADs were included in the study cohort. The selection procedures were shown in Fig. 1.

We likewise extracted the comparison cohort from the LHID2005. We randomly selected one comparison subject for every study subject, matched by sex, age (18 ~ 30, 30 ~ 39, 40 ~ 49, 50 ~ 59, 60 ~ 69, and >69 years), and index year. While for the study cohort, the year of the index date was the year in which the study subjects received their first diagnosis of ADs, for the comparison cohort, the year of the index date was simply a matched year in which comparison subjects had a medical utilization. We assigned the date of their first use of medical services occurring during that matched year as the index date for the comparison cohort. We ensured that no selected comparison subjects had ever received a diagnosis of ADs since initiation of the Taiwan NHI program in 1995. We also assured that no selected comparison subjects had ever received a diagnosis of major psychiatric disorders, substance-related disorder, or any type of cancer prior to the index date.

Thereafter, each patient in the study (*n* = 117,206) was individually tracked for a 5-year period from their index date to identify whether or not they had received a diagnosis of urological cancer (ICD-9-CM code185 ~ 189). The diagnoses of urological cancers have been confirmed by the registry of catastrophic illness. For the occurrence of cancer to be reported in the registry, histological confirmation is required.

### Statistical analysis

All statistical analyses were performed by the SAS statistical package (SAS System for Windows, vers. 8.2, Cary, NC, USA). We used the Kaplan-Meier method to estimate the 5-year cancer-free survival rate and the log-rank test to examine differences in the risks of urological cancer xbetween these two cohorts. We used stratified Cox proportional hazard regressions, stratified by sex, age group, and index year to calculate the cancer hazard for the study cohort relative to the comparison cohort. We adjusted for demographic and socioeconomic characteristics, medical comorbidities, and alcohol and tobacco use in the regression model. The variables of socioeconomic characteristics included monthly income (New Taiwan (NT)$0 ~ 15,840, NT$15,841 ~ 25,000, ≥NT$25,001; the average exchange rate in 2011 was US$1 ≈ NT$29), geographical location (northern, central, eastern, and southern Taiwan), and urbanization level of each subject’s residence (5 levels, with 1 indicating the most urbanized and 5 the least urbanized). NT$15,840 was used as the first cut-off value as it is the government-stipulated minimum wage for full-time employees in Taiwan. Medical comorbidities selected in this study were hypertension, diabetes, obesity, alcohol abuse, and tobacco use disorder. All medical comorbidities were defined by using ICD-9-CM codes in LHID2005. In addition, we censored subjects who died from non-cancer causes during the 5-year follow-up period in the regression model. Of the sampled subjects, 3749 died from non-cancer causes, including 2051 from the study cohort (3.5% of study cohort) and 1698 from the comparison cohort (2.9% of the comparison cohort). The differences were considered significant with a two-sided p value of ≤0.05.

## Results

Of the 117,206 study subjects, the mean age was 51.0 years (standard deviation, 16.5 years), while they were 51.1 years for the study cohort and 50.9 years for the comparison cohort (*p* = 0.209). Table 1 shows the demographic and socioeconomic characteristics of subjects with and without ADs. The overwhelming majority (61.8%) of study subjects were female, and over half were aged <50 years (about 57%). There were significant differences between the study and comparison cohorts in terms of monthly income, geographical location, and urbanization level of each subject’s residence. In addition, subjects with ADs had a higher prevalence of medical comorbidities of hypertension, diabetes, obesity, and alcohol and tobacco use problems than subjects without ADs (all *p* < 0.001).

**Table 1:** Subjects with anxiety disorder and a comparison group in relation to sociodemographic characteristics and medical comorbidities in Taiwan (*n* = 117,206)

Variable	Patients with anxiety disorder *n* = 58,603		Comparison cohort *n* = 58,603		*p* value
	No.	Percent	No.	Percent	
Sex					1.000
Male	22,394	38.2	22,394	38.2	
Female	36,209	61.8	36,209	61.8	
Age (years)					1.000
<40	19,722	33.8	19,722	33.8	
40 ~ 49	13,269	22.7	13,269	22.7	
50 ~ 59	10,654	18.2	10,654	18.2	
60 ~ 69	7,405	12.7	7,405	12.7	
>69	7,323	12.6	7,323	12.6	
Monthly income					<0.001
≤NT$15,840	24,159	41.2	23,299	39.8	
NT$15,841 ~ 25,000	22,503	38.4	22,108	37.7	
≥NT$25,001	11,941	20.4	13,196	22.5	
Geographic region					<0.001
Northern	25,542	43.6	27,622	47.1	
Central	16,292	27.8	13,642	23.3	
Southern	15,574	26.6	15,797	27.0	
Eastern	1,195	2.0	1,542	2.6	
Urbanization level					<0.001
1 (most urbanized)	16,858	28.8	17,685	30.2	
2	16,235	27.7	16,224	27.7	
3	8,593	14.7	9,509	16.2	
4	9,217	15.7	8,288	14.1	
5 (least urbanized)	7,700	13.1	6,897	11.8	
Obesity	670	1.1	390	0.7	<0.001
Alcohol abuse/alcohol dependence syndrome	790	1.4	69	0.1	<0.001
Tobacco use disorder	1,497	2.6	355	0.6	<0.001
Diabetes	6,611	11.3	5,483	9.4	<0.001
Hypertension	18,871	32.2	12,041	20.6	<0.001

Note: In 2011, the average exchange rate was US$1 ≈ New Taiwan (NT) $29

**Fig. 2:**
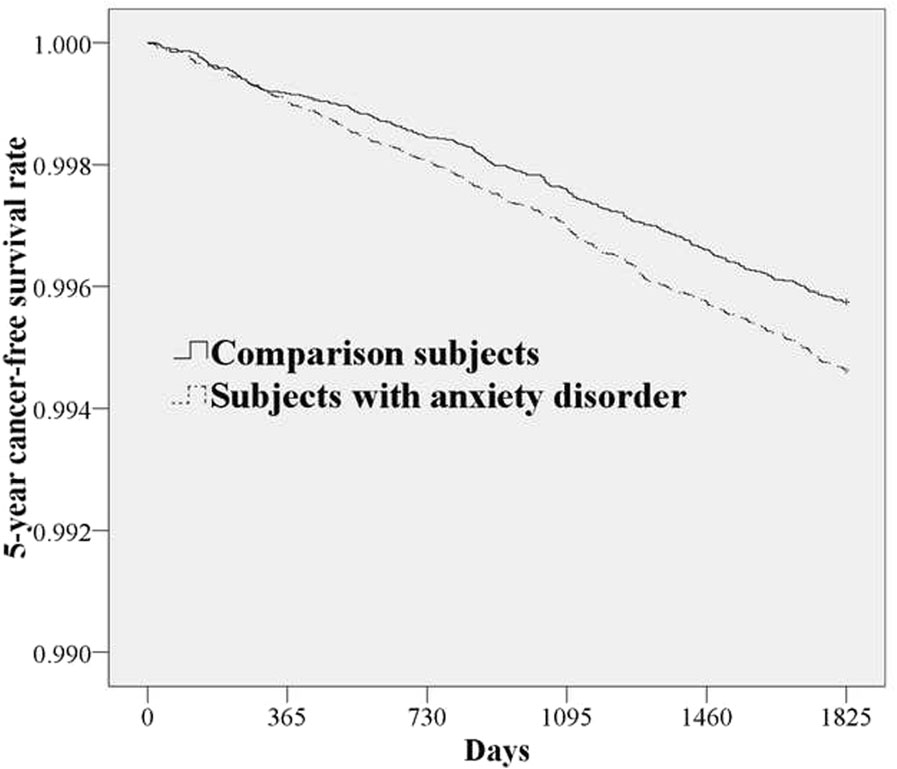
Urological cancer-free survival rates for subjects with anxiety disorder and comparison subjects

Table 2 presents the incidence rate for a new urological cancer diagnosis by cohort within the 5-year follow-up period. Of the 117,206 subjects, 391 (0.33%) received a diagnosis of urological cancer: 315 in the study cohort (0.54% of the subjects with anxiety disorder) and 76 in the comparison cohort (0.13% of subjects without anxiety disorder). The incidence rates of urological cancers during the 5-year follow-up period were 10.93 (9.76 ~ 12.21) per 10,000 person-years for the study cohort and 2.62 (2.07 ~ 3.28) per 10,000 person-years for the comparison cohort. The log-rank test revealed that subjects with ADs had a significantly lower 5-year urological cancer-free survival rate than comparison subjects (*p* = 0.005). Figure 2 presents results of the Kaplan-Meier survival analysis.

**Table 2:** Hazard ratios (HRs) and 95% confidence intervals (CIs) of urological cancer among sampled subjects during the 5-year follow-up period from the index date

Presence of urological cancer	Total sample *n* = 117,206		Patients with anxiety disorder *n* = 58,603		Comparison cohort *n* = 58,603	
	No.	Percent	No.	Percent	No.	Percent
Yes	391 (0.33)		315 (0.54)		76 (0.13)	
Incidence rate per 10,000 person-years (95% CI)	6.76 (6.11 ~ 7.47)		10.93 (9.76 ~ 12.21)		2.62 (2.07 ~ 3.28)	
Crude HR ^a^(95% CI)	–		4.16*** (3.23 ~ 5.34)		1.00	
Adjusted HR^b^ (95% CI)	–		3.67*** (2.85 ~ 4.72)		1.00	

*Notes:* ^a^Stratified Cox proportional regression stratified on patients’ sex, age group, and index year. ^b^Adjusted for patients’ monthly income, urbanization level, geographic region, hypertension, diabetes, obesity, tobacco use disorder, and alcohol abuse/alcohol dependence syndrome

***p < 0.001

Results of the stratified Cox proportional regressions (stratified on sex, age group, and index year) are also presented in Table 2. The crude hazard ratio (HR) for urological cancer during the 5-year follow-up period for the study cohort was 4.16 (95% confidence interval (CI): 3.23 ~ 5.34) with respect to the comparison cohort. After adjusting for urbanization level, monthly income, geographic region, hypertension, diabetes, obesity, and alcohol and tobacco use, and censoring cases that died from non-cancer causes, the HR for urological cancer during the 5-year follow-up period for study cohort was 3.67 (95% CI: 2.85 ~ 4.72) that of comparison patients.

Table 3 presents the proportional HRs of subgroups classified by the type of urological cancers (kidney cancer, bladder cancer, prostate cancer, and others). The adjusted HRs for kidney cancer, bladder cancer, prostate cancer, and others for subjects with ADs were 2.84 (95% CI: 1.73 ~ 4.66), 2.94 (95% CI: 1.89 ~ 4.58), 4.67 (95% CI: 3.06 ~ 7.12), and 6.75 (95% CI: 2.01 ~ 22.69), respectively, relative to those without ADs.

**Table 3:** Hazard ratios (HRs) and 95% confidence intervals (CIs) of urological cancer among sampled subjects during the 5-year follow-up period from the index date according to the type of urological cancer

Presence of urological cancer	Patients with anxiety disorder *n* = 58,603		Comparison cohort *n* = 58,603	
	No.	Percent	No.	Percent
Kidney cancer				
Yes	67 (0.11)		21 (0.04)	
Incidence rate per 100,000 person-years (95% CI)	23.20 (17.98 ~ 29.47)		7.24 (4.48 ~ 11.07)	
Adjusted HR^a^,^b^ (95% CI)	2.84*** (1.73 ~ 4.66)		1.00	
Bladder cancer				
Yes	88 (0.15)		26 (0.04)	
Incidence rate per 100,000 person-years (95% CI)	30.48 (24.45 ~ 37.55)		8.97 (5.86 ~ 13.14)	
Adjusted HR^a^,^b^ (95% CI)	2.94*** (1.89 ~ 4.58)		1.00	
Prostate cancer (men only)				
Yes	138 (0.62)		26 (0.12)	
Incidence rate per 10,000 person-years (95% CI)	12.53 (10.53 ~ 14.81)		2.35 (1.53 ~ 3.44)	
Adjusted HR^a^,^b^ (95% CI)	4.67*** (3.06 ~ 7.12)		1.00	
Others				
Yes	22 (0.04)		3 (0.01)	
Incidence rate per 100,000 person-years (95% CI)	7.62 (4.77 ~ 11.53)		1.04 (0.21 ~ 3.02)	
Adjusted HR^a^,^b^ (95% CI)	6.75** (2.01 ~ 22.69)		1.00	

*Notes:* ^a^Stratified Cox proportional regression stratified on patients’ sex, age group, and index year. ^b^ Adjusted for patients’ monthly income, urbanization level, geographic region, hypertension, diabetes, obesity, tobacco use disorder, and alcohol abuse/alcohol dependence syndrome

**p < 0.01, ***p < 0.001

The incidences of urological cancers within 5 years after the index date between patients with ADs and those without ADs stratified by sex are presented in Table 4. The adjusted HR for males with ADs was 3.82 (95% CI: 2.79 ~ 5.23) in comparison to males without ADs. In addition, the adjusted HR for females with ADs was 3.47 (95% CI: 2.26 ~ 5.31) than those females without ADs.

**Table 4:** Hazard ratios (HRs) and 95% confidence intervals (CIs) of urological cancer among sampled subjects during the 5-year follow-up period from the index date according to sex

Presence of urological cancer	Males				Females			
	Patients with anxiety disorder (*n* = 22,394)		Comparison cohort (*n* = 22,394)		Patients with anxiety disorder (*n* = 36,209)		Comparison cohort (*n* = 36,209)	
	No.	Percent	No.	Percent	No.	Percent	No.	Percent
Yes	209 (0.93)		49 (0.22)		106 (0.29)		27 (0.07)	
Incidence rate per 10,000 person-years (95% CI)	19.01 (16.52 ~ 21.77)		4.43 (3.27 ~ 5.85)		5.95 (4.87 ~ 7.19)		1.51 (0.99 ~ 2.19)	
Crude HR^a^ (95% CI)	4.28*** (3.14 ~ 5.85)		1.00		3.93*** (2.58 ~ 6.00)		1.00	
Adjusted HR^b^ (95% CI)	3.82*** (2.79 ~ 5.23)		1.00		3.47*** (2.26 ~ 5.31)		1.00	

*Notes:* ^a^Stratified Cox proportional regression stratified on patients’ age group and index year

^b^Adjusted for patients’ monthly income, urbanization level, geographic region, hypertension, diabetes, obesity, tobacco use disorder, and alcohol abuse/alcohol dependence syndrome. ***p < 0.001

## Discussion

A relationship exists between ADs and the subsequent occurrence of urological cancers, as suggested by the results herein. The findings of this study were consistent with previous reports [12, 13]. Shen and colleagues reported that male patients with generalized ADs had an increased standardized incidence ratio (SIR) for prostate cancer (2.17, 95% CI: 1.56 ~ 2.93) [13]. Since the prevalence of ADs in the general population is substantial, the observed SIR may introduce bias and cause underestimation of the true relative risk [17]. Another study by Liang and colleagues only demonstrated an increased risk of developing prostate cancer among patients with ADs older than 65 years [12]. The failure to exclude comorbid psychiatric disorders made the study group more heterogeneous which could have biased the results toward zero.

Both studies argued that the higher risk of prostate cancer could be partially explained by a surveillance bias. Patients with ADs were likely to search for medical help and thus might have received more imaging and laboratory examinations [6, 7, 18]. If true, the overall risk of cancer should also increase among patients with ADs, which was not supported by previous study results. Patients with ADs have more urological complaints [19, 20]. Liang and colleagues further validated their assumption regarding a surveillance bias by showing a higher prostate-specific antigen (PSA) screening rate among patients with ADs [12]. However, the proposed explanation is difficult to apply to the significantly increased risks of urological cancers other than prostate cancer among patients with ADs.

Another explanation stems from unhealthy lifestyles and medication use among patients with ADs. Patients with ADs tend to be overweight and physically inactive and are more likely to use alcohol and tobacco [21, 22]. In addition, a stress-prone personality and unfavorable coping styles commonly seen among patients with ADs might place them at a higher cancer risk [8]. Even so, the overall cancer risks were not consistently high in previous studies [11, 12].

In an earlier comprehensive review of the relationship between lifestyle issues and urological cancers, only strong connections of tobacco use with bladder and renal cancers were repeatedly supported by the literature [23]. Other lifestyle risk factors, if they existed, had only modest impacts on the development of urological malignancies. Furthermore, patients with ADs remained at a significantly high risk of developing all kinds of urological cancers after adjusting for tobacco use disorder in this study.

The long-term use of psychotropic medications, particularly benzodiazepine anxiolytics, is prevalent among patients with ADs. However, study results of the carcinogenic potential of benzodiazepine anxiolytics remain equivocal [24, 25]. Kao and colleagues reported that patients with a history of taking benzodiazepine were more likely to develop prostate and bladder/kidney cancers with respective HRs of 1.72 and 1.76 [24]. There is still no specific mechanisms or explanations which account for the observed increased risk, and their findings were challenged for failing to control numerous confounding factors [26].

The current study demonstrated that patients with ADs were more likely to develop prostate cancer after adjusting for available demographic, socioeconomic, and comorbid medical and substance use variables. A direct mechanism linking ADs itself to urological cancers should not be neglected.

As stated before, stress-related psychosocial factors can have adverse effects on cancer incidence and survival by disturbing functions of the immune system, thus enhancing the risk of carcinogenesis [27]. Lines of evidence suggest that persistent activation of the hypothalamic-pituitary-adrenal (HPA) axis can alter the function of the neuroendocrine system, disrupt the circadian glucocorticoid rhythm, and thus promote tumor initiation and progression [28–31]. More specifically, stress-related neurotransmitters such as serotonin were proposed as being correlated with the progression of urological cancers [32, 33].

As an important monoamine neurotransmitter in the brain, serotonin plays a crucial role in the neurobiological processing of anxiety. Dysregulation of serotonin is strongly associated with the development of ADs [34]. Serotonin also serves as a messenger that controls several processes of the body including carcinogenesis. Siddiqui and colleague found that serotonin could increase the proliferation of bladder cancer cells in vitro in a dose-dependent manner and could modify the growth inhibitory effect of doxazosin on prostate and bladder cancer cells [15, 35]. Based on relevant findings, serotonin was proposed to be a prognostic marker for urological cancer [36]. Although the majority of previous studies were conducted in vitro and clinical applications remain controversial [37]. We argue that dysregulation of the serotonin receptor pathway might contribute to the relationship between ADs and urological cancers.

Lines of evidence support that traumatic experience may alter core physiological systems and even cause carcinogenesis through DNA breakage [38]. Traumatic experience, even early childhood maltreatment, may increase the risk of ADs [39]. On the other hand, effective treatment such as psychotherapy could reverse DNA damage [40]. There could be a remote and common cause for both ADs and urological cancers. The strength of this study comes from it utilizing a large, population-based sample. Still, the results should be interpreted in light of potential methodological limitations. First of all, the use of non-standardized diagnoses made by different physicians is an inherent problem with population-based studies. By restricting our analyses to two visits with a diagnosis of ADs with at least one having been made by a board-certified psychiatrist, we enhanced the specificity of the psychiatric diagnoses. Further, we chose a broad approach to a diagnosis of ADs in order to reduce misclassification. Second, information about certain risk factors for urological cancer including environmental exposure, amount of tobacco and alcohol use, body mass index and family cancer history is not available in the database. Information on traumatic events and critical life events is also unavailable. We adjusted for the geographical location and urbanization level of each subject’s residence in the study. The diagnoses of tobacco and alcohol use disorders, which represent the most severe form of tobacco and alcohol use, were also considered in our analyses. In addition, the LHID2005 provides no information about serotonin levels or the serotonin axis. Consequently, we could not demonstrate the notion whether serotonin is important in patients with ADs developing bladder cancer. Still, a potential bias could exist. And last, a 5-year follow up period was chosen to ensure an adequate time window for the development of urological cancers among subjects at risk. Some subjects might have had a more-insidious onset and could not be identified within the 5-year follow-up period.

## Conclusions

Despite these limitations, we found that during the 5-year follow-up period, the risk of developing urological caner was much higher among patients with ADs, compared to their counterparts without ADs, and this association was totally independent from comorbid hypertension, diabetes, obesity, and registered alcohol and tobacco use disorders. Clinicians should be aware of the increased risk of developing urological cancer among patients with ADs and are encouraged not to neglect relevant urological complaints.

## Abbreviations

ADs: Anxiety disorders
HR: hazard ratio
LHID2005: Longitudinal health insurance database 2005
NHI: National health insurance
SIR: Standardized incidence ratio.
